# Validity and reliability of the Maslach Burnout Inventory-Student Survey in Sri Lanka

**DOI:** 10.1186/s40359-018-0267-7

**Published:** 2018-11-12

**Authors:** Nuwan Darshana Wickramasinghe, Devani Sakunthala Dissanayake, Gihan Sajiwa Abeywardena

**Affiliations:** 1 Department of Community Medicine, Faculty of Medicine and Allied Sciences, Rajarata University of Sri Lanka, Saliyapura, 50008 Sri Lanka; 2 Department of Community Medicine, Faculty of Medicine, University of Peradeniya, Peradeniya, 20400 Sri Lanka; 3 Teaching Hospital-Kandy, Kandy, 20000 Sri Lanka

**Keywords:** Burnout, MBI-SS, Student burnout, Collegiate cycle, Sri Lanka, Confirmatory factor analysis, Validity, Reliability

## Abstract

**Background:**

With ever increasing educational expectations and demands, burnout has emerged as a major problem negatively affecting the wellbeing of different student populations. Even though the validity of the Maslach Burnout Inventory-Student Survey (MBI-SS) is widely assessed across the globe, there is a paucity of related literature in the South Asian settings. Hence, this study was aimed at assessing the factorial structure, validity, and reliability of the MBI-SS among collegiate cycle students in Sri Lanka.

**Methods:**

The pre-tested Sinhala version of the MBI-SS was administered to a sample of 194 grade thirteen students in the Kurunegala district, Sri Lanka. The construct validity of the MBI-SS was assessed using multi-trait scaling analysis and confirmatory factor analysis (CFA), while reliability was assessed using internal consistency and test-retest reliability, which was assessed after an interval of two weeks.

**Results:**

CFA revealed that the three-factor model of the MBI-SS fitted the data better than the one-factor and the two-factor model. Only one item (item 13) was identified as having poor psychometric properties. A modified version of the MBI-SS, with item 13 deleted, emerged as an acceptable fitting model with a combination of absolute, relative and parsimony fit indices reaching desired threshold values. All three subscales show high internal consistency with Cronbach’s α coefficient values of 0.837, 0.869, and 0.881 and test-retest reliability was high (*p* < 0.001).

**Conclusions:**

The Sinhala version of the 15-item MBI-SS is a valid and a reliable instrument to assess the burnout status among collegiate cycle students in Sri Lanka. The Sinhala version of the 15-item MBI SS, due to its brevity, ease of administration, and sound psychometric properties, could be used as an effective screening tool to assess student burnout at the school level.

## Background

In the context of ever increasing educational expectations and demands having negative repercussions on mental wellbeing of student populations, exploration of the problem of burnout has become a timely need across the globe. However, defining burnout as a clinical entity has been a controversial issue throughout its course. Yet, the most widely used definition is the three-dimensional concept of burnout that was described by Maslach, Jackson, and Leiter [1].

The virtual use of the Maslach Burnout Inventory (MBI) at the budding stages of burnout research has led to the artefactual notion that burnout was exclusively found among the human services professionals [2]. The introduction of the Maslach Burnout Inventory-General Survey (MBI-GS) has paved the way to expand the horizons of burnout research outside the human services, as its dimensions are defined more generally and do not refer to working with recipients [3].

The concept of student burnout has been in the limelight with the introduction of the Maslach Burnout Inventory-Student Survey (MBI-SS) by Schaufeli et al. [2]. Though students are not employed in a work setting, the core structured and obligatory activities they are involved in, such as attending classes and finishing assignments, are targeted at the ultimate objective of passing examinations [4]. Hence, from a psychological perspective, their coercive core activities can be considered as ‘work’ [5].

In accordance with the original definition of burnout, Schaufeli et al. [2] have defined student burnout as, “a three-dimensional syndrome that is characterised by feelings of exhaustion due to the demands of studying, a cynical attitude of withdrawal and detachment, and reduced professional efficacy regarding academic requirements”. According to that definition [2], Exhaustion (EX) can be defined as feelings of strain, particularly chronic fatigue resulting from overtaxing work. Cynicism (CY) is manifested in an indifferent or a distal attitude toward work in general, a loss of interest in one’s work and not seeing it as meaningful. Reduced Professional Efficacy (PE) refers to diminished feelings of competence as well as less successful achievements and to lack of accomplishment in one’s work.

Though various study instruments have been used to assess burnout among student populations, the first reported literature pertaining to the invention of a specific measure to assess student burnout is the invention of MBI-SS by Schaufeli et al. [2]. Since then, MBI-SS has been cited as the most widely used research instrument to assess burnout in different student populations across the globe [6–8]. MBI-SS is one of the latest additions to the family of inventories of MBI. This inventory is a modified form of the MBI-GS. The MBI-SS, which is a self-administered questionnaire, consists of 16 items representing the three dimensions of student burnout. The three-factor conceptualisation of the MBI-SS has been confirmed in different student populations in different countries [2, 5, 9–12]. However, hitherto, there is no published literature pertaining to the validity of MBI-SS in the South Asian context.

Though not widely used as the MBI-SS, the School Burnout Inventory, which consists of three dimensions, developed by Salmela-Aro et al. [13], the two-factor Oldenburg Burnout Inventory student version developed by Campos et al. [14] and the Copenhagen Burnout Inventory-Student version developed by Campos et al. [15] have been used to assess the concept of student burnout.

Even though a plethora of research have been conducted among different student populations pertaining to burnout across the globe, the published literature on the topic in the South Asian context is scanty. In Sri Lanka, the period of general education comprises all grades from grade one to thirteen in the school system and the collegiate cycle in the education system consists of grade twelve and grade thirteen. At the end of the collegiate cycle, grade thirteen students sit for the General Certificate of Examination (GCE) Advanced Level, which is the national level selection examination for state university admissions. Studies conducted on assessing mental health issues among Sri Lankan collegiate cycle students reveal that the prevalence of mental health problems such as depression and anxiety are high and further evidence suggests that symptoms are mainly attributable to examination induced stress [16]. In addition, the findings of a national survey revealed that nearly one in five adolescents in schools appear to have clinically relevant mental health problems [17] and approximately one third of adolescents had indicated that they felt pressurized due to the parents’ and teachers’ expectations of higher academic performance [18]. Against the backdrop of high prevalence of mental health problems in students, exploring the concept of student burnout is extremely important and a timely, as burnout directly assesses the psychological well-being in relation to academic endeavours. However, owing to the absence of a validated instrument to assess burnout in the Sri Lankan context, this important research area is not widely explored.

In this background, the present study was designed to assess the construct validity and reliability of the MBI-SS among collegiate cycle students and to explore the applicability of the three-factor model of the MBI-SS in the Sri Lankan context.

## Methods

### Study design and setting

This school-based, cross-sectional validation study was conducted in the Kurunegala district, North Western province, Sri Lanka. The study was conducted from May 2014 to April 2015 in three Sinhala medium government schools in the Kurunegala district. All these three schools have students studying in all four collegiate cycle subject streams, viz., Science, Arts, Commerce, and Technology.

### Participants

Three classes each were selected from the three selected schools and this selection represented both male and female students studying in all four subject streams. The total number of students participated in the study was 194 and the response rate was 100.0%. The majority of the participants were females (*n* = 107, 55.2%). The mean age of the sample was 18.3 years (SD = 0.43 years). The number of students in the Science, Arts, Commerce, and the Technology streams were 78 (40.2%), 60 (30.9%), 41 (21.2%), and 15 (7.7%) respectively.

### Measures

In the 16-item MBI-SS, which is a self-administered questionnaire, five items are targeted at identifying EX, five items are targeted at identifying CY, and six items are targeted at identifying PE. A seven-point rating scale is used to assess the frequency in which the respondents experience feelings related to each dimension and this rating scale ranges from 0 (never) to 6 (every day). According to the scores of each dimension, the high scores on EX and CY and low scores on PE are indicative of burnout.

The forward-backward translation method was used to translate the 16-item MBI-SS to Sinhala. This forward-backward translation method is a widely accepted method for cross-cultural adaptation of study instruments [19–21]. The method included, forward translation, backward translation, and pre-testing and cognitive interviewing. Two bilingual translators, who are fluent in Sinhala and English, independently translated the questionnaire into Sinhala while ensuring semantic equivalence, conceptual equivalence, and normative equivalence. To produce a synthesis of the two forward translations, an independent reviewer, who is fluent in both languages, reviewed both translations together with the original English version. Any discrepancies and ambiguities between the translated versions and any deficiencies compared to the original English version were resolved by consensus. The synthesised forward translated version was agreed upon for the backward translation. Two sworn language translators, who were totally blind to the original English version of the MBI-SS, independently translated the synthesised forward translation of MBI-SS back into English, without referring to the original version. Pre-testing of the synthesised forward translation of the MBI-SS was conducted among a sample of 25 grade thirteen students who were studying in schools outside the study setting. This sample consitsted of both male and female students studying in all four Advanced Level subject streams.

Face, content, and the consensual validity were assessed in order to appraise the judgemental validity of the questionnaire. A multi-disciplinary panel of experts representing the fields of psychiatry, psychology, public health, teaching, student counseling, and medical education assessed the consensual validity of the MBI-SS Sinhala version. The expert panel assessed each item of the questionnaire on its relevance in assessing burnout among grade thirteen students, appropriateness of the wording used, and acceptability in the local context for assessing burnout among grade thirteen students by using a rating scale of 0 to10, in which 0 being strong disagreement and 10 being strong agreement. In addition to rating of each item, the panelists were asked to make additional remarks related to the phrasing of items. Except for the item 13 stating, “I just want to get my work done and not be bothered”, all other items had a median score more than 7 for all the aspects. Based on the compiled rating scores and the comments, it was decided to include all 16 items in the synthesised forward translation of MBI-SS with suggested modifications, to be considered for confirmatory factor analysis (CFA).

### Procedure

Ethical approval for this study was obtained from the Ethics Review Committee of the Faculty of Medicine and Allied Sciences, Rajarata University of Sri Lanka (Reference no: ERC/2014/057). Administrative clearance for the study was obtained from the Provincial Director of Education, North Western province, and the principals of the selected three schools. Data collection was done according to the logistic convenience of the schools to minimise the disturbance to the routine academic and other endeavours. Prior to data collection, informed written consent was obtained from all the participants and each participant was given the Sinhala version of MBI-SS to be filled independently. Confidentiality of the data collected and the anonymity of the participants were maintained. To assess the test-retest reliability of the study instrument, two weeks after the initial date of data collection, the same questionnaire was re-administered to students in a grade thirteen class who were included in the initial data collection.

### Data analysis

Multi-trait scaling analysis and CFA were carried out on the scores obtained from the study participants to assess the construct validity of the MBI-SS. In relation to the scores of the data set, as low scores on PE subscale are indicative of burnout, reversed PE (rPE) scores were used for further statistical analysis.

Prior to performing statistical analyses, the suitability of the data set was assessed for any violations of assumptions demanded by the analytical techniques and the dataset did not violate the assumptions related to the level of measurement, related pairs, independence of observations, normality (using histograms and standardised skewness and kurtosis values), linearity (using bivariate scatter plots), outliers, and multicollinearity. Since the sample size was 194 and there were 16 observed variables, the ratio of observations to variables was approximately 12.1:1; hence, the sample size was adequate to conduct the analysis [22].

### Multi-trait scaling analysis

Multi-trait scaling analysis was conducted using the SPSS version 17.0. Item-scale correlations were analysed and item-convergent and item-discriminant validity were assessed. In assessing item-convergent validity, a stringent criterion of correlation of 0.40 or greater between an item and its own subscale was considered as a success for assessing [23]. Items which correlated significantly higher (more than 1.96 standard errors) with its own subscale than with the other two subscales were considered as scaling successes in assessing item-discriminant validity.

### CFA

In relation to factorial validity, as the three-factor model of MBI-SS is well established and substantiated by numerous research findings, CFA was employed to assess the extent to which underlying three-factor model was replicated in the observed data using the analytic software Linear Structural Relations (LISREL) version 9.1. The structure of the MBI-SS was evaluated based on a variety of fit indices, including absolute fit indices, relative fit indices and parsimony fit indices. Satorra-Bentler scaled chi-square test, Root Mean Square Error of Approximation (RMSEA), Goodness-of-Fit Index (GFI), Adjusted Goodness-of-Fit Index (AGFI), and Standardised Root Mean Square Residual (SRMR) were used as the absolute fit indices. Comparative Fit Index (CFI) and Non-Normed Fit Index (NNFI) were used as the relative fit indices, while Parsimony Goodness-of-Fit Index (PGFI) and Parsimonious Normed Fit Index (PNFI) were used as the parsimony fit indices.

The analysis was conducted in two steps. In the first step, following models were assessed.

- One-factor model: All 16 items of MBI-SS were loaded on to one latent factor.
- Two-factor model: Items measuring EX (five items) and measuring CY (five items) were loaded on to a single latent factor and items measuring rPE (six items) were loaded on to a different latent factor.
- Three-factor model: Items measuring EX, CY, and rPE were loaded on to three separate latent factors.

In the second step, specification search for the three-factor model was carried out considering the psychometric properties evaluated for the items in previous validity assessment methods, changes made to the three-factor model in the previous studies, and also the suggestions for modifications offered by LISREL analysis. In this step, modified three models of the original three-factor model were compared with each other.

- Model 1: Owing to the complexity of covariance structure models and correlational data, it is likely that model modifications would substantially improve the fit of the model to the data [24]. Hence, six correlated error terms were added to the three-factor model as per the suggestions for modifications offered by LISREL analysis.
- Model 2: Previous studies regarding the factorial validity of MBI-SS have removed the item 13, as it was found to be ambivalent and thus unsound [2, 25]. In appraising consensual validity of the items of MBI-SS, this item received low median rating scores by the multi-disciplinary panel of experts. Furthermore, item 13 did not yield a scaling success at item-discriminant validity in multi-trait scaling analysis. Hence, it was decided to delete this item from MBI-SS and the modified model was evaluated in CFA.
- Model 3: Though the model evaluated in the previous step yielded improvement in several fit indices, it was decided to incorporate six correlated error terms as per the suggestions for modifications offered by LISREL analysis.

### Assessment of reliability

In order to assess the reliability or the consistency of information gathered by the Sinhala version of MBI-SS, two methods, viz., internal consistency and test-retest reliability were employed.

Test-retest reliability was assessed by administering the Sinhala version of MBI-SS after a gap of two weeks in a sub-sample of participants enrolled in the study.

## Results

### Descriptive statistics of the MBI-SS scores

Scoring of the MBI-SS Sinhala version was carried out according to the instructions provided in the MBI manual [1]. The manual recommends reporting means and SD of each subscale. Furthermore, it recommends computing the average rating scores across the items within each of the three subscales. The scores of PE subscale, which is inversely associated with burnout showed a higher, mean item score (4.34, SD = 1.27) compared to the other two subscales. Descriptive statistics of the MBI-SS subscales are given in Table 1.

**Table 1:** Descriptive statistics of the MBI-SS subscale scores among grade thirteen students (*n* = 194)

Subscale	Mean total score	SD	Mean item score	SD
EX	11.66	6.29	2.33	1.26
CY	9.89	4.87	1.98	0.98
PE	26.07	7.61	4.34	1.27
rPE	9.93	7.61	1.66	1.27

### Multi-trait scaling analysis

Results of the multi-trait scaling analysis conducted on MBI-SS validation study are summarised in Table 2. The item-convergent validity and the item-discriminant validity of each item were assessed by item-scale correlations. Item-convergent validity was supported if an item correlates substantially (a corrected correlation of 0.40 or more) with the scale it is hypothesised to represent. Hence, except for the item13, for all other items, item-convergence was confirmed. Item discrimination was supported if the correlation between an item and the subscale that it is hypothesised to measure was significantly larger (more than 1.96 standard errors) than the correlations of that item with other subscales. Except for the item13, all item-scale correlations for other items emerged as scaling successes. Hence, according to multi-trait scaling analysis, except for the item 13, item-convergent validity and item-discriminant validity were confirmed for other 15 items in the MBI-SS.

**Table 2:** Item-scale correlations of the MBI-SS, item-convergent and item-discriminant validity (*n* = 194)

Item	EX score	CY score	rPE score	Standard error (SE)	Cut-off value (−1.96 SE)	Scaling success
EX1	0.785	0.499	0.594	0.045	0.697	Success
EX2	0.764	0.466	0.564	0.047	0.672	Success
EX3	0.779	0.511	0.598	0.045	0.690	Success
EX4	0.791	0.501	0.627	0.044	0.704	Success
EX6	0.786	0.537	0.634	0.045	0.698	Success
CY8	0.668	0.762	0.683	0.048	0.667	Success
CY9	0.623	0.810	0.695	0.042	0.727	Success
CY13	0.687	0.366	0.579	0.067	0.234	Not success
CY14	0.657	0.838	0.744	0.039	0.761	Success
CY15	0.642	0.810	0.688	0.042	0.727	Success
PE5	0.593	0.487	0.710	0.051	0.610	Success
PE7	0.690	0.617	0.841	0.039	0.764	Success
PE10	0.677	0.685	0.829	0.040	0.749	Success
PE11	0.615	0.678	0.827	0.041	0.747	Success
PE12	0.534	0.552	0.787	0.044	0.699	Success
PE16	0.530	0.669	0.753	0.047	0.659	Success

### CFA

Model fit statistics in relation to absolute, relative, and parsimony fit indices of the first step, i.e. one-factor, two-factor, and three-factor models are summarised in the Table 3. According to Satorra-Bentler scaled Chi-square test, none of the factor models tested fit the data well (*p* < 0.001). However, χ^2^ statistic is sensitive to sample size and it nearly always rejects the model when large samples are used [26, 27]. Hence, the results were interpreted in conjunction with other model fit indices. The RMSEA values did not meet the threshold value of a good model fit for any of the three models tested, though the value for the three-factor model showed relative improvement. Both GFI and AGFI values were indicative of model improvement in the three-factor model in comparison to other two models at sub-optimal level. Furthermore, SRMR value was within the desirable range only for the three-factor model.

**Table 3:** Model fit statistics of one-factor, two-factor and three-factor models of the MBI-SS

Model	Absolute fit indices							Relative fit indices		Parsimony fit indices	
	χ^2^	df	p	RMSEA	GFI	AGFI	SRMR	CFI	NNFI	PGFI	PNFI
One-factor model	252.58	90	0.000	0.097	0.838	0.784	0.0513	0.971	0.967	0.628	0.820
Two-factor model	280.37	103	0.000	0.095	0.832	0.779	0.0510	0.972	0.968	0.630	0.821
Three-factor model	258.56	101	0.000	0.090	0.850	0.798	0.0498	0.975	0.971	0.631	0.808

*χ*^*2*^ Satorra-Bentler scaled Chi-square test (desired value *p* > 0.05), *RMSEA* Root Mean Square Error of Approximation (desired value < 0.08), *GFI* Goodness-of-Fit Index (desired value > 0.9), *AGFI* Adjusted Goodness-of-Fit Index (desired value > 0.9), *SRMR* Standardised Root Mean Square Residual (desired value < 0.05), *CFI* Comparative Fit Index (desired value > 0.95), *NNFI* Non-Normed Fit Index (desired value > 0.95), *PGFI* Parsimony Goodness-of-Fit Index (desired value > 0.5), *PNFI* Parsimonious Normed Fit Index (desired value > 0.5)

All relative (CFI & NNFI) and parsimony (PGFI & PNFI) fit indices showed values above the desired levels for all the three models tested and the three-factor model yielded comparatively better results. Hence, it was concluded that the three-factor model showed improvement compared to other two models. However, the overall fit of the three-factor model warranted further improvement.

The model fit statistics of the second step related to the specification search are summarised in Table 4. Irrespective of the model modifications, χ^2^ test remained significant. However, introduction of six correlated error terms into the three-factor model of MBI-SS, improved the RMSEA value and it yielded desired value compatible with a good model fit (0.068). In spite of the improvement in GFI and AGFI indices, the values remained below the desired value of a good model fit (0.892 and 0.846 respectively). The three-factor model with item 13 deleted also yielded similar results. However, except for SRMR of 0.0470, the other indices did not improve beyond the desired values. In contrast, the model with item 13 deleted and six correlated error terms added, showed substantial improvement in RMSEA value. Not only the value was 0.064, but also the upper bound of 90% CI was also below the desired value of 0.08. The GFI was 0.911, which is also beyond the desired value.

**Table 4:** Model fit statistics in specification search of the three-factor model of the MBI-SS

Model	Absolute fit indices							Relative fit indices		Parsimony fit indices	
	χ^2^	df	p	RMSEA	GFI	AGFI	SRMR	CFI	NNFI	PGFI	PNFI
Three-factor model	258.56	101	0.000	0.090	0.850	0.798	0.0498	0.975	0.971	0.631	0.808
Three-factor model + correlated error terms	179.57	95	0.000	0.068	0.892	0.846	0.0407	0.987	0.983	0.623	0.770
Three-factor model with item 13 deleted	211.84	87	0.000	0.086	0.869	0.819	0.0470	0.978	0.973	0.630	0.798
Three-factor model with item 13 deleted + correlated error terms	146.38	81	0.000	0.064	0.911	0.868	0.0403	0.988	0.974	0.615	0.752

*χ*^*2*^ Satorra-Bentler scaled Chi-square test (desired value p > 0.05), *RMSEA* Root Mean Square Error of Approximation (desired value < 0.08), *GFI* Goodness-of-Fit Index (desired value > 0.9), *AGFI* Adjusted Goodness-of-Fit Index (desired value > 0.9), *SRMR* Standardised Root Mean Square Residual (desired value < 0.05), *CFI* Comparative Fit Index (desired value > 0.95), *NNFI* Non-Normed Fit Index (desired value > 0.95), *PGFI* Parsimony Goodness-of-Fit Index (desired value > 0.5), *PNFI* Parsimonious Normed Fit Index (desired value > 0.5)

All relative (CFI and NNFI) and parsimony (PGFI and PNFI) fit indices showed values above the desired levels for all the three models tested. Hence, the results are suggestive that the three modified models of the three-factor model showed superior fit to data in comparison with the original three-factor model.

**Fig. 1:**
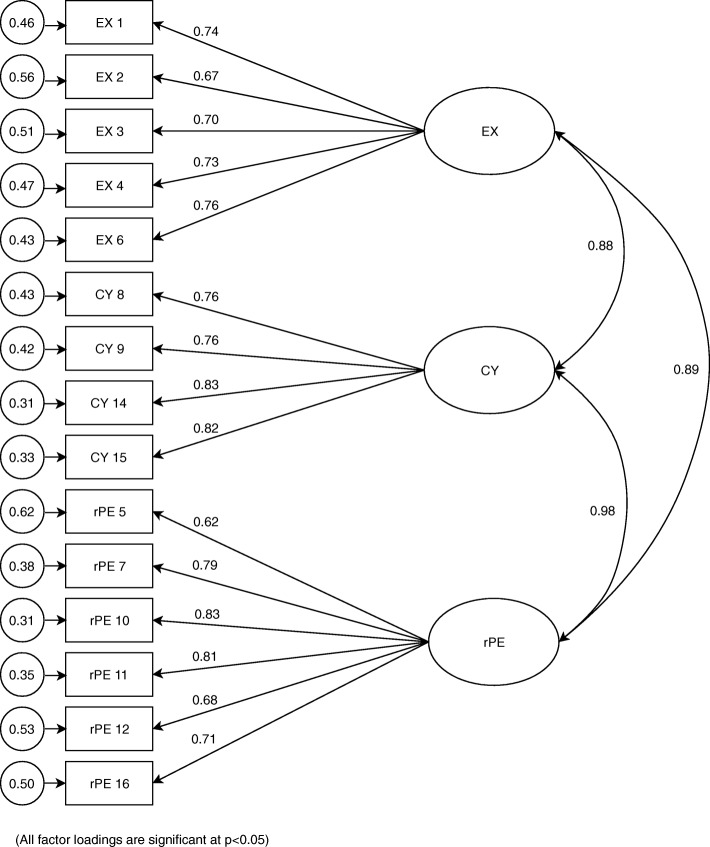
Standardised parameter estimate for the factor structure of the MBI-SS with item 13 deleted. (EX: Exhaustion; CY: Cynicism, rPE: reversed Professional Efficacy)

Following the specification search, it was evident that the addition of error covariances to the model resulted in improvement of the model fit. Considering the fact that no model fits real-world phenomena exactly and the problems encountered with addition of error covariances to the model, the three-factor model with item13 deleted was considered as an acceptable model, which fits the data. This conclusion is substantiated by having a combination of fit indices representing all three categories, which reached desired threshold values. The resultant standard parameter estimates for the modified factor structure is given in Fig. 1 and in this model, all the factor loadings of this model were statistically significant (*p* < 0.05). Furthermore, all the items had factor loadings larger than 0.6 from its own latent factor.

### Reliability

#### Internal consistency

Internal consistency was assessed by calculating Cronbach’s α coefficient for each subscale of the MBI-SS. Validated MBI-SS consisted of five items measuring EX subscale, four items measuring CY subscale and six items measuring rPE subscale. The impact of each item on the related subscale was assessed by computing Cronbach’s α when the respective item is deleted. None of the items included in the analysis showed α values greater than the final α value. Hence, all the items were retained in the analysis. According to the analysis, all three subscales showed high internal consistency with Cronbach’s α coefficient values of 0.837, 0.869, and 0.881 for EX, CY, and rPE subscales respectively.

#### Test-retest reliability

The data from a group of 22 students collected two weeks after the initial administration of the MBI-SS were assessed for the test-retest reliability of the instrument and the test-retest reliability assessment revealed strong, positive correlations for each of the three subscales of MBI-SS. For the EX, CY, and rPE subscales, the correlation coefficients were 0.858, 0.910, and 0.890 respectively. The correlation coefficients were statistically significant at *p* < 0.001.

## Discussion

The concept of student burnout has been explored across different student populations representing varying educational contexts [2, 5, 9–12]. However, the novelty of the concept and the absence of a proper assessment tool have hindered the exploration of the concept in many of the South Asian countries including Sri Lanka.

The present study was designed with the objective of validating the Sinhala version of the MBI-SS among Sri Lankan collegiate cycle students. Hence, a cross sectional design deemed appropriate for this purpose. Review of literature in relation to student burnout has demonstrated that the concept of burnout shows heterogeneity across different educational contexts. Hence, it is pertinent to select a specific student population to whom a common educational context is applicable. Taking this issue into consideration, the students in the collegiate cycle, who were studying in grade thirteen, were selected to minimise the heterogeneity in relation to their academic endeavours. In addition, the study sample was selected to represent both male and female students studying in all four subject streams. Three Sinhala medium government schools were selected considering the logistic feasibility to conduct clinical interviews by the Consultant Psychiatrist, the ease of accruing a relatively large number of students on a given date of data collection and having satisfactory infrastructure facilities in the schools to arrange suitable places for data collection and conducting clinical interviews. The study sample was similar to the national statistics related to the sex distribution and the sample distribution pattern with regard to subject streams was not very different from that of the country profile, with Arts, Science and Commerce being the main subject streams and a relatively small percentage of students studying in the Technology subject stream. Furthermore, as mentioned above, the sample size of the study deemed adequate to conduct a validation study [22].

The forward-backward translation method, which is a widely accepted method for cross-cultural adaptation of study instruments [19–21], was employed in the translation of the study instrument. During the process, particular emphasis was given to ensure semantic equivalence, conceptual equivalence and normative equivalence. This was achieved by conducting this process in conjunction with language experts and the technical experts. Furthermore, a multi-disciplinary panel of experts representing many important fields related to student burnout has assessed the judgmental validity of the questionnaire. Expect for the item 13, all the other items were found to have high median rating scores. Item 13 (“I just want to get my work done and not be bothered”) was identified as ambivalent. Though it is an item that reflects a negative attitude, it could also be interpreted as a positive attitude by those who would like to successfully complete academic endeavors without making them to bother their lives. Similarly, this item was identified as having poor psychometric properties by some other researchers largely owing to its ambivalent nature [2, 5, 25, 28]. The studies that have used 15-item MBI-SS, have also omitted this specific item from the study instrument [11, 29]. However, the reasons for the omission have not been stated.

Multi-trait scaling analysis was used to assess the hypothesised scale structure of the MBI-SS as the primary step in analysing whether the set of items in MBI-SS can be appropriately combined into summated rating scales [23]. In multi-trait scaling analysis, except for the item 13, item-convergent validity and item-discriminant validity were confirmed for other 15 items in the MBI-SS. The ambivalent nature of the item 13 may have resulted in not having satisfactory item-convergent and item-discriminant validity in multi-trait scaling analysis.

CFA is considered as a viable method of assessing the construct validity of study instruments. CFA necessitates a strong priori theory underlying the measurement model before analysing data [30]. Additionally, CFA is often used in data analysis to examine the expected level of causal connections between variables [31]. Since the tri-dimensional structure of the MBI-SS has been widely established in literature, CFA was employed to assess the construct validity of the MBI-SS in the present study. Since there is no consensus as to what category of model fit indices are to be used in assessing the model fit, a combination of absolute fit indices, relative fit indices, and parsimony fit indices were used in the present study for that purpose [32].

The results of the CFA revealed that the three-factor model fitted the data set better than the one-factor and the two-factor models. This finding is congruent with the findings of several other researchers who have tested the CFA of one-factor model and the three-factor model [1]. Analysis revealed that, the values for all absolute fit indices, relative fit indices, and parsimony fit indices of three-factor model were better than those of one-factor and two-factor models. However, among the absolute fit indices, only SRMR had reached the stipulated cut-off value (< 0.05).

Since the fit indices of the three-factor model showed room for further improvement, specification search for the three-factor model was carried out. To overcome the psychometric limits of the MBI, several procedures have been highlighted in the literature. These methods include, allowing correlated error terms, allowing items to load on more than one factor, eliminating items, and increasing or decreasing the number of factors [33]. In the present study, the specification search was carried out considering the psychometric properties evaluated for the questionnaire items in previous validity assessment methods. In relation to that, since item 13 had received low median rating scores in assessing the judgmental validity and since the item had unsatisfactory results at multi-trait scaling analysis, a modified three-factor model with item 13 deleted was tested in CFA. Furthermore, the suggestions for modifications in relation to adding correlated error terms offered by LISREL were considered in the specification search.

The modified three-factor model with item 13 deleted proved to be a better model fit to the data in comparison to the original three-factor model. Additionally, the modified three-factor models with addition of correlated error terms had revealed superior fit to data in comparison with the original three-factor model. The possible reasons behind this phenomenon are, the existence of random measurement errors or unmeasured variables underlying the items. However, this improvement in model fit is at the expense of lack of generalisability of the findings. According to MacCallum et al. [24], the specification search process is inherently susceptible to capitalisation on chance, owing to the potential role of idiosyncratic characteristics of the sample influencing the particular modifications.

Even though the modified three-factor models with addition of correlated error terms had revealed superior fit to data in comparison with the original three-factor model, they had not shown substantial improvement in the model fit when compared with the modified three-factor model with item 13 deleted. Considering the fact that no model fits real-world phenomena exactly and the problems encountered with addition of correlated error terms to the model, the three-factor model with item 13 deleted was considered as an acceptable model, which fits the data. This conclusion is substantiated with having a combination of fit indices representing all the three categories, which reached desired threshold values (RMSEA = 0.080, SRMR = 0.0470, CFI = 0.978, NNFI = 0.973, PGFI = 0.630, PNFI = 0.798). This finding is consistent with other studies conducted to assess the validity of the MBI-SS [2, 5, 34]. Even though, few other MBI-SS validation studies had revealed that 15-item MBI-SS showed acceptable fit to data, they have not specified which item had been deleted from the original 16-item version of the MBI-SS [35, 36].

Assessment of reliability of the Sinhala version of the MBI-SS was done by assessing the internal consistency and the test-retest reliability. Internal consistency revealed that, all three subscales of burnout were having high Cronbach’s α coefficient values. This finding is consistent with findings of other studies conducted across the globe, involving different language versions of the MBI-SS. The internal consistency was revealed as high in Portuguese version [2, 11, 29], Spanish version [2], Dutch version [2], Chinese version [5], Turkish version [34–36], and Persian version [37] of the MBI-SS.

The present study revealed that the test-retest correlation coefficients were high for each of the three subscales of the Sinhala version of the MBI-SS. The study conducted by Kutsal and Bilge [34] revealed very high coefficient values for all three subscales in a sample of Turkish high school students after a gap of three weeks.

Given that the results generated from factor analysis are often sample specific [38], generalisability of the present study findings to other populations should be done with caution, considering the variations in educational and cultural contexts. Furthermore, selecting the three Sinhala medium government schools considering the logistic feasibility is another study limitation, which affects the generalisability of the study findings. Even though the total sample size was adequate to conduct factor analysis, it is important to note the relatively small number of study participants in different subject streams, which highlights the need for future studies involving multi-group analyses.

## Conclusions

The present study confirms the three dimensional structure of the student burnout concept. The Sinhala version of the 15-item MBI-SS is a valid and a reliable instrument to assess the burnout status among collegiate cycle students in Sri Lanka.

The Sinhala version of the 15-item MBI-SS, due to its brevity, relative ease of administration, and sound psychometric properties, could be used as an effective screening tool for the assessment of burnout at the school level. It will allow identification of the affected students at early stages, which is important in effective secondary prevention, and identification of vulnerable students, which is imperative for the primary prevention.

Moreover, given that the three-factor structure of the MBI-SS has been established in the Sri Lankan context strengthening the evidence base for the relevance and the applicability of the concept of student burnout in the South Asian context, future research could be conducted involving different South Asian student populations.

## Abbreviations

AGFI: Adjusted Goodness-of-Fit Index
CFA: Confirmatory Factor Analysis
CFI: Comparative Fit Index
CY: Cynicism
EX: Exhaustion
GCE: General Certificate of Examination
GFI: Goodness-of-Fit Index
LISREL: Linear Structural Relations
MBI: Maslach Burnout Inventory
MBI-GS: Maslach Burnout Inventory-General Survey
MBI-SS: Maslach Burnout Inventory-Student Survey
NNFI: Non-Normed Fit Index
PE: Professional Efficacy
PGFI: Parsimony Goodness-of-Fit Index
PNFI: Parsimonious Normed Fit Index
RMSEA: Root Mean Square Error of Approximation
rPE: reversed Professional Efficacy
SD: Standard Deviation
SRMR: Standardised Root Mean Square Residual
